# Removal of large fibrotic bladder blood clots using prostatic tissue morcellator under real-time ultrasound guidance

**DOI:** 10.3389/fsurg.2022.889529

**Published:** 2022-09-05

**Authors:** Ronghua Wu, Yonggang Shang, Xing Liu, Wei Chen, Shanhong Yi

**Affiliations:** ^1^ Department of Urology, Xinqiao Hospital of Army Medical University, Chongqing, China; ^2^ Reproductive Medicine Center, the First Affiliated Hospital of Chongqing Medical University, Chongqing, China

**Keywords:** ultrasound guidance, prostatic tissue morcellator, bladder blood clot, removal, application

## Abstract

**Objective:**

Large fibrotic bladder blood clots are difficult to treat *via* conventional methods. Hence, we investigated the safety and reliability of real-time ultrasound guidance combined with prostate tissue morcellator in the removal of large fibrotic bladder blood clots in this study.

**Methods:**

We chose 9 patients with large fibrotic bladder blood clots who were treated in our department from January 2019 to December 2020. Under the condition that conventional methods were ineffective in removing the bladder blood clot, real-time ultrasound guidance combined with a prostatic tissue morcellator was used to remove the large fibrotic bladder blood clot through the steps of positioning, breaking, adjusting repositioning and recrushing. After removal, the bipolar electrocautery was replaced to stop bleeding of the bladder mucosa.

**Results:**

All patients successfully underwent the operation. After the blood clot was removed, the bladder mucosa was examined. There was no damage to the bladder mucosa or muscle layer. The urine was clear at the end of the procedure with slow irrigation, and no bleeding was found again.

**Conclusion:**

Real-time ultrasound guidance combined with a prostate tissue morcellator was a safe, effective and quick method for the removal of large fibrotic bladder blood clots.

## Introduction

As a result of trauma, surgery and other factors leading to blood accumulation in the bladder, hematuria leading to clot retention is a common urological emergency. Once the bladder distends and catheter gets blocked, patient has abdominal distension, severe pain with bladder spasms and is in severe discomfort (1). If neglected, can cause bladder overdistension or bladder rupture and would be a serious threat to the patient's life and health (2).

Reasonable and effective removal of blood clots in the bladder is the primary task to solve this part of urological emergency (3). Fresh bleeding can be resolved by replacing the urinary catheter and washing under a cystoscope (4). However, for the slow accumulation of blood or rapid haemorrhage in the bladder, the urinary catheter is repeatedly blocked and forms a large organized bladder blood clot. The conventional treatment methods, such as using a catheter or ellik evacuator for removal, are characterized by poor efficacy, difficult suction, unclear field of vision, long operation time and vulnerability to accidental damage (5, 6). Therefore, it is necessary to perform a new method of surgical treatment to deal with this urological emergency.

In recent years, based on the development of holmium laser enucleation of prostate, a prostate tissue morcellator has been developed, which can quickly break and suck out the large lobe prostate tissue, shorten the operation time and it is safe and reliable (7). However, in patients with large prostate especially with diffuse bleeding, the vision may be unclear leading to increased risk of bladder mucosal injury during morcellation (8). For such situations, a recent study has demonstrated the improved safety and efficiency of ultrasound-guided morcellation (9).

According to the above principles and experiences, under the real-time guidance of ultrasound, we used a prostatic tissue morcellator to evacuate the blood clot, and improve vision so that hemostasis could be achieved. Nine patients were successfully treated, which is reported in this study.

## Methods

### Clinical data

**Figure 1:**
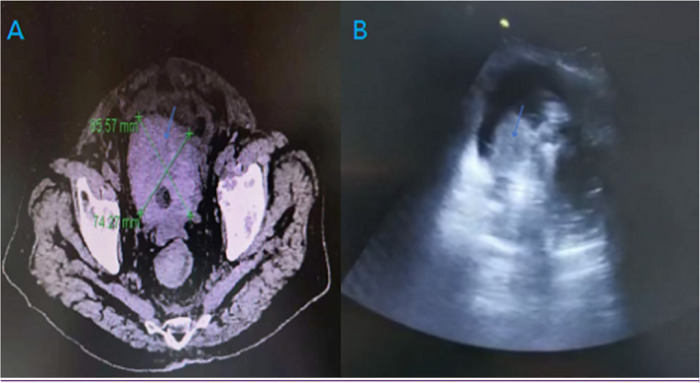
Imaging manifestations of large blood clot in bladder under CT scanning and ultrasound [(**A**): CT, (**B**) ultrasound; blue arrow: blood clot].

Nine patients presented with neglected blood clot retention in bladder because of a variety of reasons. All patients had lower abdominal distension, dysuria, urinary catheter blockage and repeated suction failure. A blood clot was confirmed by ultrasonography and CT scan. The imaging manifestations of large blood clot in bladder under CT scanning and ultrasound were presented in Figure 1. Their average age was 70.66 ± 19.65 years. The maximum diameter of the blood clot in the bladder was 9.5 cm, and the minimum was 6.5 cm. The average volume of the bladder blood clot was 205.94 ± 107.49 ml. The average onset time of urinary catheter obstruction was 42.44 ± 15.06 h. The basic characteristics of the included subjects are presented in Table 1.

**Table 1:** The basic characteristics of the included subjects.

Case	Age (Years)	Primary disease	Detection method	Clot size (cm)	Clot volume (cm^3^)	Duration of disease (Hours)	Operating Time (Mmins)	Conservative methods
1	37	Dangerous Placenta Previa	Ultrasound	7.9*8.0*8.0	262.91	18	35	Catheter aspiration
2	87	Prostate cancer	CT	8.5*7.5*7.0	232.05	23	42	Eillk
3	91	Bladder cancer	CT	5.7*3.4*5.0	50.39	45	51	Eillk
4	79	TURP	Ultrasound	11.5*9.6*7.0	401.86	65	40	Eillk and catheter aspiration
5	93	TURP	Ultrasound	6.9*7.8*6.0	167.91	45	53	Catheter aspiration
6	65	TURP	Ultrasound	5.8*5.7*6.3	108.30	56	36	Catheter aspiration
7	45	Hemorrhagic cystitis	CT	9.6*7.0*8.2	286.54	48	52	Catheter aspiration
8	72	Cystolithotripsy	CT	7.8*7.0*8.0	227.1	34	45	Eillk
9	67	Renal tumor	CT	8*0.4.0*7.0	116.48	48	48	Eillk and catheter aspiration

### Equipment setup

**Figure 2:**
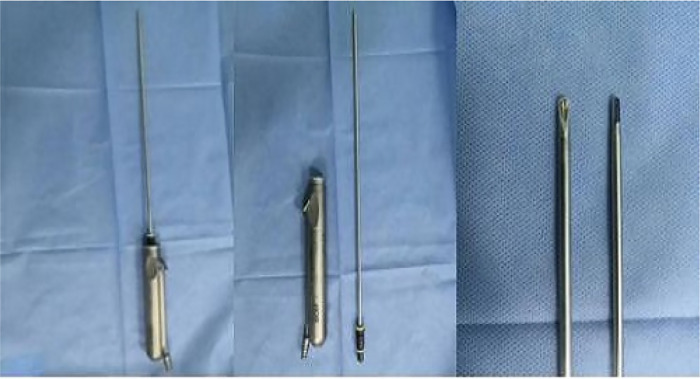
A photograph of the morcellator.

In this study, we uesed a prostate tissue morcellator (China-made Great White Shark Tissue Shredder) to remove blood clots from the bladder. A photograph of the morcellator was presented in Figure 2.

### Operation methods and techniques used

**Figure 3:**
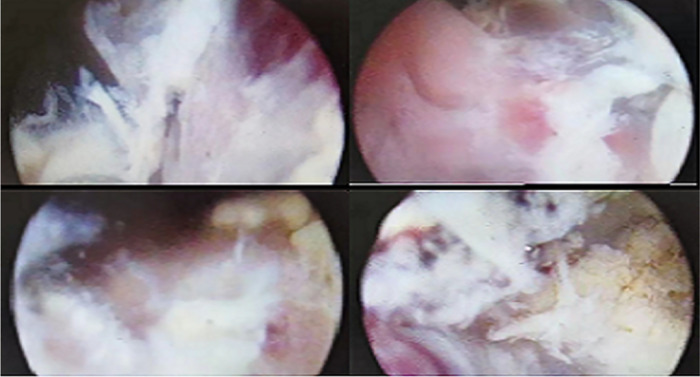
The fibrotic bladder blood clots under cystoscope.

Under general anasthesia, in lithotomy position after sterile draping, ultrasonographic evaluation of the bladder was done to confirm the clot and to rule out bladder perforation. The prostate tissue morcellator was placed in the bladder and under real-time ultrasound and cystoscopic guidance clot evacuation by morcellator was started. The photograph of fibrotic bladder blood clot under cystoscope was presented in Figure 3.

**Figure 4:**
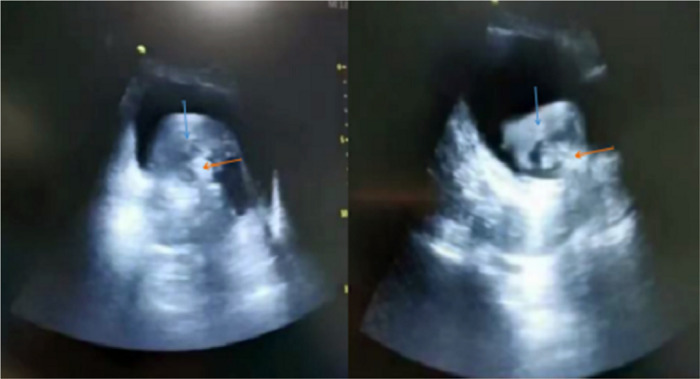
The morcellator in the centre of the blood clots under ultrasound guidance (blue arrow: blood clot; Red arrow: morcellator).

Under real-time ultrasound guidance, the knife head was first placed in the centre of the blood clot. We adjusted the ultrasonic scanning section, and it was repeatedly confirmed that the knife head of the prostate tissue crusher was located in the centre of the blood clot. The cutter head was upwards, the method of point stepping was adopted, each step 3 times, stop 1 time, and adjust the suction gear at the same time to ensure the consistency of real-time water inlet and outlet speed. After removing the central part of the blood clot, the front end of the cutter head was gradually moved in the direction of the remaining clot taking precaution that the cutter head is away from the bladder mucosa. With the gradual removal of blood clots, the visual field became clear, once the vision became clear the terminal small free floating clots were removed under cystoscopic vision. Finally hemostasis was achieved using bipolar cautery. The photograph of morcellator in the centre of the blood clots under ultrasound guidance was presented in Figure 4. For the parts that were easy to bleed, we used the bipolar electrocautery and gradually removed the blood clots. After haemostasis, we closed the water inlet, observed the visual field and water colour, and checked the whole bladder mucosa, bilateral ureteral openings and bladder triangle. When there was no obvious active bleeding and all bladder mucosa was visible, the operation was ended.

## Results

The operation was successfully completed in all cases, all bladder blood clots were removed, and there was no bladder mucosal injury caused by the prostatic tissue morcellator. The washing colour of all patients was clear after the operation. The average operation time is 44.6 min.

## Discussion

Bladder bleeding is a common emergency in the department of urology. For bladder blockage caused by acute bleeding, there are many methods to remove bladder blood clots, including replacing urinary catheters and increasing the speed of flushing fluid (10). However, for chronic bleeding, blood clots gradually increase, blood clot organization occurs in the later stage; conventional removal methods have poor efficacy, and patients who undergo such conventional treatments continue to have symptoms such as lower abdominal pain, which is more difficult to treat.

At present, the most commonly used method to remove bladder blood clots is endoscopic blood clot removal and manual bladder irrigation (4, 11), however, there were some disadvantages, such as low suction efficiency and unclear vision. This method might cause bladder mucosal damage and bladder perforation, prolong the operation time and increase the risk of infection (12, 13). One study reported that a tissue pulverizer could be used to remove bladder blood clots. However, when cleaning the large fibrotic clot filling the whole bladder cavity, the inlet and outlet water could not maintain a balance, the treatment effect was poor, and there was a certain risk when the blood clot was broken in a turbid field of vision (14).

Our treatment method has a distinct advantage in difficult situation of neglected large clot retention. As we perform clot evacuation by prostate tissue morcellator under ultrasound control, the crusher is always kept in the center of the blood clot and there is no over distension of the bladder. This avoids the risk of bladder mucosal injury and bladder perforation. We found this procedure safe and rapid reducing the stress on the treating surgeon. Our study was similar to a research suggested that ultrasound guidance combined with tissue morcellator could be an additional tool to utilize during difficult cases when cystoscopic visualization during prostate morcellation was limited (9).

The disadvantage of this method is that it requires the cooperation of both a radiologist and urologist. Radiologist need to track the cutter head orientation of the prostate tissue crusher in real time. In addition, in the early stage of operation, when the field of vision is unclear, it is necessary to manually control the water inflow to prevent bladder rupture and other injuries caused by excessive pressure. It requires the cooperation of many people to complete the whole operation. Additionally, our study is a single arm observational study with a small sample size. A larger multi-institutional randomized controlled trial is important.

## Conclusion

Under real-time guidance ultrasound, we changed the direction and depth of the cutter head of the prostatic tissue morcellator and controlled the breaking rhythm, which is a safe, effective and quick method to address the large fibrotic bladder blood clot.

## Data Availability

The original contributions presented in the study are included in the article/Supplementary Material, further inquiries can be directed to the corresponding author/s.
